# Outcomes after aortic aneurysm repair in patients with history of cancer: a nationwide dataset analysis

**DOI:** 10.1186/s12893-020-00754-3

**Published:** 2020-05-01

**Authors:** Sanghyun Ahn, Jin-Young Min, Hyunyoung G. Kim, Hyejin Mo, Seung-Kee Min, Sangil Min, Jongwon Ha, Kyoung-Bok Min

**Affiliations:** 1 Division of Vascular Surgery, Department of Surgery, Seoul National University College of Medicine, Seoul, Republic of Korea; 2 Institute of Health and Environment, Seoul National University, Seoul, Republic of Korea; 3 Department of Family and Community Medicine, University of Texas Health Sciences Center at San Antonio, San Antonio, TX USA; 4 Department of Preventive Medicine, Seoul National University College of Medicine, 103 Daehak-ro, Jongno-gu, Seoul, 110-799 Republic of Korea

**Keywords:** Abdominal aortic aneurysm, Cancer, Mortality

## Abstract

**Background:**

Synchronous cancer in patients with abdominal aortic aneurysm (AAA) increases morbidity and mortality after AAA repair. However, little is known about the impact of the history of cancer on mortality after AAA repair.

**Methods:**

Patients with intact AAA who were treated with endovascular aneurysm repair or open surgical repair were selected from the Health Insurance and Review Assessment data in South Korea between 2007 and 2016. Primary endpoints included the 30- and 90-day mortality and long-term mortality after AAA repair. The Cox proportional hazards models were constructed to evaluate independent predictors of mortality.

**Results:**

A total of 1999 patients (17.0%, 1999/11785) were diagnosed with cancer prior to the AAA repair. History of cancer generally had no effect in short-term mortality at 30 and 90 days. However, short-term mortality rate of patients with a history of lung cancer was more than twice that of patients without it (3.07% vs. 1.06%, *P* = 0.0038, 6.14% vs. 2.69%, *P* = 0.0016). Furthermore, the mortality rate at the end of the study period was significantly higher in AAA patients with a history of cancer than in those without a history of cancer (21.21% vs. 17.08%, *P* < .0001, HR, 1.31, 95% CI, 1.17–1.46).

**Conclusions:**

The history of cancer in AAA patients increases long-term mortality but does not affect short-term mortality after AAA repair. However, AAA repair could increase both short- and long-term mortality in patients with lung cancer history, and those cases should be more carefully selected.

## Background

An asymptomatic abdominal aortic aneurysm (AAA) is usually found incidentally during workup for other clinical problems or on ultrasound during regular check-ups. Because cancer patients often undergo periodic imaging studies with computed tomography scan or ultrasound examinations, they have an increased diagnosis of AAA [1]. Therefore, physicians could encounter situations where the patients with AAA have a history of cancer [2]. However, there is limited information on how the history of malignancy affects the decision and short- and long-term outcome of AAA repair.

Many studies have analyzed the effect of AAA repair in patients with synchronous malignancy and showed increased short-term mortality and morbidity after both endovascular aortic repair (EVAR) and open surgical repair (OSR) [3]. In addition, patients with intra-abdominal tumors have increased perioperative mortality after OSR of AAA, and the history of intra-abdominal procedures may hinder OSR due to the development of adhesions [4]. Thus, EVAR may be the treatment of choice in cancer patients with suitable anatomy while its adequacy is yet to be determined.

Despite improvements in short-term outcome after AAA repair, long-term survival is still not favorable [5–7]. End-stage renal disease (ESRD), chronic obstructive pulmonary disease (COPD), AAA diameter, and age at the time of surgery are known and unmodifiable risk factors affecting the survival after AAA repair [8]. A history of cancer can also affect the long-term survival of patients undergoing AAA repair [9]. Therefore, the risk and benefit of AAA repair should be carefully evaluated according to the patient’s life expectancy and potential risk factors [10].

The purpose of our study was to investigate the effect of cancer history on the outcome of AAA repair. In this retrospective study using big data, we first determined the incidence and types of cancer history in patients who underwent AAA repair. We then evaluated how cancer history affects short- and long-term mortality after AAA repair.

## Methods

### Patient and public involvement

The Health Insurance Review and Assessment Service (HIRA) reviews the accuracy of claims and renders reimbursement decisions for the National Health Insurance, covering approximately 98% of the entire South Korean population. We used the claims data of HIRA, comprised of detailed health care service information, including diagnosis, treatment, procedure, surgical history, and prescription drugs, along with the International Classification of Diseases, Tenth Revision (ICD-10) diagnosis codes [11].

From the HIRA data, a study cohort of patients diagnosed with AAA (I71.01, I71.3, I71.4, I71.5, I71.6, I71.8, I71.9) between January 1, 2007 and December 31, 2016 was created. Of the 79,880 eligible patients, 68,095 who were diagnosed with ruptured AAA (I71.3, I71.5, I71.8), untreated by EVAR or OSR, or had an unverified date of death were excluded. The death of a patient was designated as an “event,” and living patients were censored on December 31, 2016. The final sample included 11,785 patients with intact AAA treated by EVAR or OSR.

Patients with a history of cancer were defined as those diagnosed with malignant neoplasms (ICD-10 C00–97) more than two times prior to the EVAR or OSR. The main outcome measure was the all-cause mortality at 30 days, 90 days, and at the end of the study period following AAA repair in patients with and without a cancer history.

### Variables of interest

The demographic variables included age (5-year increments: < 65, 65–69, 70–74, 75–79, 80–84, 85–89, versus ≥90) and sex (male versus female). Health insurance coverage was categorized into the National Health Insurance Program, a mandatory enrollment scheme, and the Medical Aid Program for low-income citizens. The hospitals where EVAR or OSR were performed ranged from small general hospitals with 100–300 beds to mid-sized general hospitals with 300–1000 beds to tertiary research university hospitals with over 1000 beds. The hospital locations were divided into urban and rural areas. The comorbidities were selected using the Charlson Comorbidity Index (CCI), a method of categorizing comorbidities based on the ICD-10 codes, and grouped based on the CCI scores 0–1, 2, and ≥ 3 [12]. Specific comorbidities included hypertension (ICD-10: I10), diabetes mellitus (ICD-10: E10, E11), myocardial infarction (ICD-10: I21, I22), and end-stage renal diseases (ICD-10: N18.5).

### Statistical analysis

The statistical differences in the patient characteristics based on the history of cancer were computed using the Chi-square test. We compared the prevalence of all-cause mortality in AAA patients with and without a cancer history. All-cause mortality was calculated at 30 days, 90 days, and at the end of the study period after AAA repair. We used the Kaplan-Meier curves to depict the cumulative incidence of all-cause mortality. The statistical comparison between the survival of AAA patients with and without cancer history was performed using the log-rank test.

To estimate the effect of cancer on death in AAA patients treated by EVAR or OSR, we conducted univariate (crude) and multivariable (adjusted) Cox-proportional hazards regression analyses with respect to the occurrence of all-cause deaths. Hazard ratios (HRs) and 95% confidence intervals (CIs) for the outcome were calculated in AAA patients with and without a cancer history. The Cox-proportional regression models were adjusted for the demographic variables (i.e., age and sex) in Model 1 and then further adjusted for all covariates (i.e., CCI, insurance type, hospital type, regional area, and comorbidities) in Model 2. All analyses were performed using the SAS 9.4 software (SAS Institute, Cary, NC, USA), and the statistical significance level was set at α = .05.

## Results

A total of 11,785 patients with intact AAA were included in this study, of which 1999 patients (17.0%) had preexisting cancer. The distribution of different types of malignancy is shown in Table 1. There were 1515 intra-abdominal and 1067 digestive cancers. Stomach cancer (21.5%) was the most commonly diagnosed cancer, followed by colorectal (19.1%), prostate (18.4%), and lung cancer (11.5%).

**Table 1:** Types of malignancy

	ICD code	Number	%
**All sites**		2315	
**Oral cavity & pharynx**	C00–14	26	1.12
**Digestive system**		1067	46.09
Esophagus	C15	76	
Stomach	C16	432	
Small intestine	C17	9	
Colon and rectum	C18, 19, 20	385	
Anus, anal canal, & anorectum	C21	3	
Liver & intrahepatic bile duct	C22	105	
Gallbladder & other biliary	C23, 24	46	
Pancreas	C25	40	
Other digestive organs	C26	6	
**Thorax (including heart)**		282	12.18
Lung & bronchus	C34	228	
Other respiratory organs	C30–33, 35–39	56	
**Bones & joints**	C40–41	3	0.13
**Skin & soft tissue**	C43–49	50	2.16
**Breast**	C50	26	1.12
**Genital system**		384	16.59
Uterine cervix	C53	11	
Uterine corpus	C54, 55	1	
Ovary	C56	1	
Other genital, female	C51, 52, 57, 58	0	
Prostate	C61	365	
Testis	C62	4	
Other genital, male	C60, 63	4	
**Urinary system**		206	8.9
bladder	C67	114	
Kidney & renal pelvis	C64, 65	90	
Ureter & other urinary organs	C66, 68	16	
**Eye & orbit**	C69	1	0.04
**Brain & other nervous system**	C70–72	4	0.17
**Endocrine system**		58	2.51
Thyroid	C73	57	
Other endocrine	C74, 75	1	
**lymphoid neoplasms**		50	2.16
Lymphoma	C81–86, 88	28	
Myeloma	C90	10	
Leukemia	C91–95	11	
Other lymphoid	C96	2	
**Other & unspecified primary sites**	C76–80, 97	158	6.83

Table 2 compares the characteristics of intact AAA patients with and without a cancer history. The AAA patients with cancer history were more likely to be older, male, with higher CCI scores, seek care in the urban area and have certain comorbidities (diabetes mellitus and dyslipidemia) compared to those without a cancer history. There was no difference in insurance type, hospital type, and comorbidities (hypertension, myocardial infarction, and ESRD) between the two groups.

**Table 2:** Characteristics of intact AAA patients with or without cancer

	All patients (*n* = 11,785)	with cancer (*n* = 1999)		without cancer (*n* = 9786)		*p*-value
Age (year)						
< 65	2755	265	(9.62)	2490	(90.38)	<.0001
65–69	2190	374	(17.08)	1816	(82.92)	
70–74	2746	531	(19.34)	2215	(80.66)	
75–79	2410	513	(21.29)	1897	(78.71)	
80–84	1263	243	(19.24)	1020	(80.76)	
85–89	368	63	(17.12)	305	(82.88)	
≥ 90	53	10	(18.87)	43	(81.13)	
Sex						
Male	9685	1798	(18.56)	7887	(81.44)	<.0001
Female	2100	201	(9.57)	1899	(90.43)	
Insurance type						
Medical aid program	859	137	(15.95)	722	(84.05)	0.4111
National health insurance	10,926	1862	(17.04)	9064	(82.96)	
Hospital type						
Tertiary hospital	8864	1501	(16.93)	7363	(83.07)	0.8855
General/small hospital	2921	498	(17.05)	2423	(82.95)	
Regional area						
Urban area	8865	1581	(17.83)	7284	(82.17)	<.0001
Rural area	2920	418	(14.32)	2502	(85.68)	
Charlson Comorbidity Index						
0–1	3400	520	(15.29)	2880	(84.71)	0.0003
2	3252	531	(16.33)	2721	(83.67)	
≥ 3	5133	948	(18.47)	4185	(81.53)	
Comorbidities						
Hypertension	9791	1677	(17.13)	8114	(82.87)	0.2881
DM	4050	809	(19.98)	3241	(80.02)	<.0001
Dyslipidemia	8204	1468	(17.89)	6736	(82.11)	<.0001
MI	910	171	(18.79)	739	(81.21)	0.1259
ESRD	160	31	(19.38)	129	(80.63)	0.4129

*AAA* Abdominal aortic aneurysm, *DM* Diabetes mellitus, *MI* Myocardial infarction, *ESRD* End-stage renal disease

Data are presented as number (%)

*p*-value was calculated using the chi-square test

Table 3 shows the prevalence of all-cause mortality of AAA patients with and without a cancer history. In AAA patients treated by EVAR or OSR, the prevalence of all-cause mortality in the patients diagnosed with cancer was higher than in those without the diagnoses at the end of the study period (21.21% vs. 17.08%; P = <.0001). Total mortality was significantly higher in AAA patients with cancer history than in those without after EVAR (22.09% vs. 17.51%; *P* = 0.0010) and OSR (21.65% vs. 16.87%; *P* = 0.0054). Regardless of EVAR or OSR, there were no differences in 30-day and 90-day mortality between the two groups (*P* > .05).

**Table 3:** All-cause mortality rates in intact AAA patients with or without cancer

All-cause mortality		with cancer		without cancer		*p*-value
**Patients with intact AAA treated by EVAR or OSR (*n*** **= 11,785)**						
30-day mortality	*Censored*	1980	(99.05)	9676	(98.88)	0.4967
	*Event*	19	(0.95)	110	(1.12)	
90-day mortality	*Censored*	1931	(96.60)	9529	(97.37)	0.0537
	*Event*	68	(3.40)	257	(2.63)	
Total mortality	*Censored*	1575	(78.79)	8115	(82.92)	<.0001
	*Event*	424	(21.21)	1671	(17.08)	
**Patients with intact AAA treated by EVAR (*n*** **= 7903)**						
30-day mortality	*Censored*	1560	(99.11)	6259	(98.89)	0.4534
	*Event*	14	(0.89)	70	(1.11)	
90-day mortality	*Censored*	1521	(96.63)	6150	(97.17)	0.2570
	*Event*	53	(3.37)	179	(2.83)	
Total mortality	*Censored*	1242	(78.91)	5221	(82.49)	0.0010
	*Event*	332	(22.09)	1108	(17.51)	
**Patients with intact AAA treated by OSR (*n*** **= 3882)**						
30-day mortality	*Censored*	420	(98.82)	3837	(98.84)	0.9719
	*Event*	5	(1.18)	45	(1.16)	
90-day mortality	*Censored*	410	(96.47)	3789	(97.60)	0.1053
	*Event*	15	(3.53)	93	(2.40)	
Total mortality	*Censored*	333	(78.35)	3227	(83.13)	0.0054
	*Event*	92	(21.65)	655	(16.87)	

*AAA* Abdominal aortic aneurysm, *EVAR* Endovascular aneurysm repair, *OSR* Open surgical repairs

**Fig. 1:**
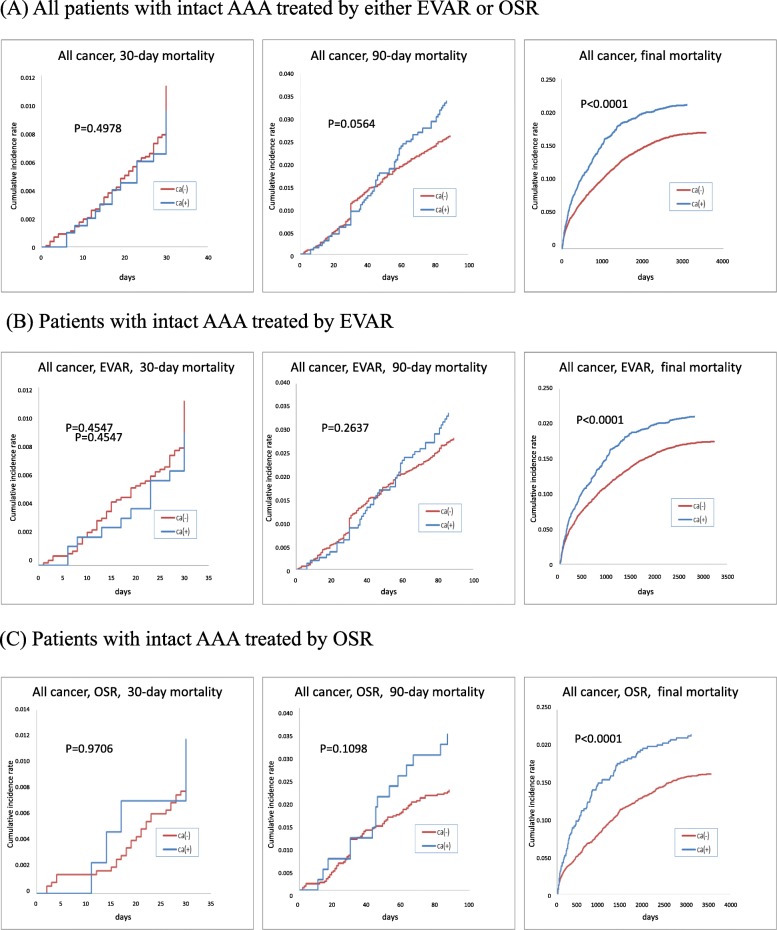
Kaplan-Meier survival curves for all-cause mortality in intact AAA patients by the presence of cancer: **a** All patients with intact AAA treated by EVAR or OSR, **b** Patients with intact AAA treated by EVAR, and **c** Patients with intact AAA treated by OSR

Fig. 1 represents the Kaplan-Meier curves for the cumulative incidence of overall mortality by a history of cancer. The AAA patients with cancer history had significantly higher mortality at the end of the study period (*P* < .0001) than in those without a cancer history. In contrast, 30-day and 90-day mortality rates in patients who underwent AAA repair did not differ based on the presence of cancer history.

Table 4 shows the HRs for all-cause mortality of intact AAA patients with a cancer history. Among the AAA patients treated by EVAR or OSR, patients with cancer history showed significantly increased HRs for the total mortality (HR = 1.50; 95% CI, 1.35–1.67) than those without. After adjusting for potential covariates, the estimate decreased but remained for total mortality (HR = 1.31; 95% CI, 1.17–1.46). The risk of total mortality was similar in AAA patients treated with EVAR (adjusted HR = 1.28; 95% CI, 1.13–1.46) and those treated with OSR (adjusted HR = 1.33; 95% CI, 1.06–1.66). On the contrary, HRs for 30-day and 90-day mortality were not significant whether the patients with AAA had cancer or not.

**Table 4:** Hazard ratio for all-cause mortality associated with the presence of cancer in patients with intact AAA

All-cause mortality	Crude HR (95% CIs)		Adjusted HR (95% CIs)^*^			
			Model 1		Model 2	
**Patients with intact AAA treated by EVAR or OSR (*n*** **= 11,785)**						
30-day mortality	0.85	(0.52–1.38)	0.83	(0.50–1.36)	0.80	(0.49–1.31)
90-day mortality	1.30	(0.99–1.69)	1.20	(0.92–1.58)	1.16	(0.89–1.53)
Total mortality	1.50	(1.35–1.67)	1.31	(1.18–1.46)	1.31	(1.17–1.46)
**Patients with intact AAA treated by EVAR (*n*** **= 7903)**						
30-day mortality	0.80	(0.45–1.43)	0.82	(0.46–1.46)	0.80	(0.45–1.43)
90-day mortality	1.19	(0.88–1.62)	1.17	(0.86–1.60)	1.14	(0.83–1.55)
Total mortality	1.40	(1.24–1.58)	1.29	(1.14–1.46)	1.28	(1.13–1.46)
**Patients with intact AAA treated by OSR (*n*** **= 3882)**						
30-day mortality	1.02	(0.40–2.58)	0.96	(0.37–2.45)	0.87	(0.34–2.24)
90-day mortality	1.57	(0.90–2.73)	1.35	(0.77–2.36)	1.28	(0.73–2.24)
Total mortality	1.55	(1.24–1.94)	1.32	(1.06–1.65)	1.33	(1.06–1.66)

*AAA* Abdominal aortic aneurysm

^*^Model 1: Adjusted for age and sex

Model 2: Further adjusted for insurance type (National Health Insurance or Medical Aid Program), hospital type (tertiary hospital or general/small hospital), regional area (urban or rural), CCI (0–1, 2, or ≥ 3), and comorbidities (hypertension, diabetes mellitus, dyslipidemia, myocardial infarction, or end-stage renal disease)

We also calculated HR according to the history of intra-abdominal and digestive cancers and lung cancers. There was a significant increase in HRs for long-term but not short-term mortality in patients with intra-abdominal and digestive cancer history (Tables 5 and 6). Interestingly, HRs for 30- and 90-day mortality as well as long-term mortality were significantly increased in patients with lung cancer history (Table 7).

**Table 5:** Hazard ratio for all-cause mortality associated with the presence of intra-abdominal cancer in intact AAA patients

All-cause mortality	Crude HR (95% CIs)		Adjusted HR (95% CIs)^*^			
			Model 1		Model 2	
**Patients with intact AAA treated by EVAR or OSR (*n*** **= 11,785)**						
30-day mortality	0.82	(0.47–1.44)	0.81	(0.46–1.41)	0.79	(0.45–1.38)
90-day mortality	0.98	(0.70–1.36)	1.18	(0.97–1.45)	0.87	(0.62–1.21)
Total mortality	1.41	(1.25–1.59)	1.21	(1.07–1.36)	1.21	(1.07–1.37)
**Patients with intact AAA treated by EVAR (*n*** **= 7903)**						
30-day mortality	0.67	(0.33–1.33)	0.68	(0.34–1.36)	0.66	(0.33–1.33)
90-day mortality	0.86	(0.59–1.25)	0.83	(0.57–1.22)	0.80	(0.55–1.18)
Total mortality	1.34	(1.17–1.53)	1.21	(1.05–1.38)	1.21	(1.05–1.39)
**Patients with intact AAA treated by OSR (*n*** **= 3882)**						
30-day mortality	1.43	(0.57–3.63)	1.38	(0.70–2.73)	1.28	(0.50–3.31)
90-day mortality	1.39	(0.72–2.67)	1.19	(0.61–2.31)	1.14	(0.59–2.22)
Total mortality	1.37	(1.06–1.78)	1.13	(0.87–1.47)	1.16	(0.89–1.51)

*AAA* Abdominal aortic aneurysm

^*^Model 1: Adjusted for age and sex

Model 2: Further adjusted for CCI (0–1, 2, or ≥ 3) insurance type (National Health Insurance or Medical Aid Program), hospital type (tertiary hospital or general/small hospital), regional area (urban or rural), and comorbidities (hypertension, diabetes mellitus, dyslipidemia, myocardial infarction, or end-stage renal disease)

**Table 6:** Hazard ratio for all-cause mortality associated with the presence of digestive cancer in patients with intact AAA

All-cause mortality	Crude HR (95% CIs)		Adjusted HR (95% CIs)^*^			
			Model 1		Model 2	
**Patients with intact AAA treated by EVAR or OSR (*n*** **= 11,785)**						
30-day mortality	0.84	(0.44–1.61)	0.85	(0.44–1.62)	0.82	(0.43–1.57)
90-day mortality	1.10	(0.76–1.58)	1.04	(0.72–1.50)	1.00	(0.69–1.45)
Total mortality	1.45	(1.26–1.66)	1.30	(1.13–1.49)	1.31	(1.14–1.50)
**Patients with intact AAA treated by EVAR (*n*** **= 7903)**						
30-day mortality	0.84	(0.41–1.76)	0.88	(0.42–1.82)	0.87	(0.21–3.59)
90-day mortality	1.06	(0.71–1.59)	1.05	(0.70–1.58)	1.03	(0.69–1.54)
Total mortality	1.38	(1.19–1.61)	1.30	(1.11–1.51)	1.31	(1.12–1.52)
**Patients with intact AAA treated by OSR (*n*** **= 3882)**						
30-day mortality	0.87	(0.21–3.59)	0.89	(0.21–3.68)	0.55	(0.13–2.24)
90-day mortality	1.06	(0.43–2.62)	1.02	(0.41–2.51)	1.05	(0.55–2.00)
Total mortality	1.34	(0.97–1.85)	1.17	(0.85–1.62)	1.21	(0.87–1.67)

*AAA* Abdominal aortic aneurysm

^*^Model 1: Adjusted for age and sex

Model 2: Further adjusted for CCI (0–1, 2, or ≥ 3) insurance type (National Health Insurance or Medical Aid Program), hospital type (tertiary hospital or general/small hospital), regional area (urban or rural), and comorbidities (hypertension, diabetes mellitus, dyslipidemia, myocardial infarction, or end-stage renal disease)

**Table 7:** Hazard ratio for all-cause mortality associated with the presence of lung cancer in patients with intact AAA

All-cause mortality	Crude HR (95% CIs)		Adjusted HR (95% CIs)^*^			
			Model 1		Model 2	
**Patients with intact AAA treated by EVAR or OSR (*n*** **= 11,785)**						
30-day mortality	2.93	(1.37–6.28)	2.98	(1.39–6.41)	2.97	(1.38–6.39)
90-day mortality	2.33	(1.37–3.99)	2.24	(1.31–3.84)	2.27	(1.33–3.89)
Total mortality	2.07	(1.62–2.65)	1.90	(1.49–2.43)	1.95	(1.52–2.49)
**Patients with intact AAA treated by EVAR (*n*** **= 7903)**						
30-day mortality	2.85	(1.15–7.03)	3.05	(1.23–7.58)	3.04	(1.22–7.58)
90-day mortality	1.84	(0.94–3.58)	1.84	(0.94–3.58)	1.90	(0.97–3.71)
Total mortality	1.91	(1.44–2.54)	1.77	(1.33–2.36)	1.81	(1.36–2.41)
**Patients with intact AAA treated by OSR (*n*** **= 3882)**						
30-day mortality	3.26	(0.79–13.47)	3.08	(0.74–12.79)	3.26	(0.78–13.67)
90-day mortality	4.09	(1.66–10.07)	3.57	(1.44–8.83)	3.69	(1.48–9.19)
Total mortality	2.33	(1.44–3.77)	2.25	(1.39–3.64)	2.29	(1.41–3.71)

*AAA* Abdominal aortic aneurysm

^*^Model 1: Adjusted for age and sex

Model 2: Further adjusted for insurance type (National Health Insurance or Medical Aid Program), hospital type (tertiary hospital or general/small hospital), regional area (urban or rural), CCI (0–1, 2, or ≥ 3), and comorbidities (hypertension, diabetes mellitus, dyslipidemia, myocardial infarction, or end-stage renal disease)

## Discussion

Of the 11,785 patients surgically treated for intact AAA, 1999 (17.0%) had a history of cancer. Stomach cancer (21.5%) was the most commonly diagnosed cancer, followed by colorectal (19.1%), prostate (18.4%), and lung cancer (11.5%). History of cancer did not affect 30- and 90-day mortality after both OSR and EVAR. However, long-term mortality after AAA repair was significantly higher in patients with cancer history than in those without cancer history (21.21% vs. 17.08%, HR 1.31; 95% CI, 1.17–1.46).

### Cancer and AAA

Cancer patients are more likely to have imaging studies done during the course of treatment and thus incidentally found to have AAA. Our results showed that about 17% of patients who underwent AAA repair had a cancer diagnosis, and most of them were intra-abdominal cancers (74%). The types of cancer diagnosis in patients with AAA repair were similar to those in the general population [13, 14]. Tilson et al. showed a shared genetic link between cancer and AAA development [15]. On the other hand, more imaging tests also leads to an increased AAA discovery when considering the incidence and types of cancer.

As the progression in cancer treatment have been decreasing overall deaths related to cancer, vascular surgeons will more commonly see cases of AAA in cancer patients. Their history of cancer would become an important factor to consider in determining the treatment strategy.

### Short-term mortality

Both OSR and EVAR of the intact AAA did not increase mortality in patients with a history of cancer. In contrast, previous study by Kouvelos et al. of AAA repair in patients with synchronous malignancy showed higher mortality and morbidity [3]. Since we looked at a history of cancer rather than synchronous cancer, there could have been a longer time interval between the cancer treatment and AAA repair. By the time of AAA repair, patients may have recovered from the previous illness and regained their full body condition. Moreover, terminal cancer patients do not benefit from AAA repair and would have been excluded from the analysis.

We hypothesized that previous abdominal surgery interferes with OSR and results in a worse outcome. Additional analyses were performed with codes corresponding to intra-abdominal and digestive cancers. Interestingly, there was no difference in short-term mortality after OSR (1.12%) versus EVAR (1.12%). While EVAR is considered the preferred choice for AAA repair in patients with a history of abdominal surgeries, OSR also seems to be safe if well planned.

### Long-term mortality

Generally, elective AAA repair shows poor long-term survival despite improved short-term outcomes [5, 8]. Long-term mortality after AAA repair was significantly higher in patients with a history of cancer after adjusting age and sex, hospital type, CCI, hypertension, diabetes mellitus, dyslipidemia, myocardial infarction, and ESRD. This high mortality was expected as cancer and cardiovascular disease, including AAA, are major causes of death [2]. Our results demonstrate that a history of cancer should also be considered in clinical decision-making in AAA repair in addition to well-known risk factors, such as ESRD and COPD [16].

### Lung Cancer

Patients with a history of lung cancer had increased short-term mortality as well as long-term mortality after AAA repair. Among all cancers, lung cancer is well known to have the worst prognosis at an advanced stage in most cases. 80% of lung cancer is non-small cell type, and lobectomy or pneumonectomy is performed in operable patients [17, 18]. These surgeries often further deteriorate the patient’s condition, and AAA repair can be difficult in patients with lung cancer due to higher treatment-related mortality [19–21]. Therefore, the high mortality at 30 and 90 days suggests the need for reevaluation of current indications for AAA repair and for further research.

### Strengths & Limitations

Studies using big data are helpful in revealing an overall understanding when the specific diagnosis code is reliable, and the endpoint is well defined. In our study, HIRA data allowed analysis of a vast number of patients and specific variables, such as mortality, and ensured accuracy by using diagnosis codes.

Due to the inherent limitations of HIRA data, the cause of death, cancer stage, and types of cancer surgeries could not be determined. The cancer stage will affect long-term survival rather than a short-term result. When we analyzed the cause of death based on patient code at the finial hospitalization, the patients with cancer history had cancer-related code as a major diagnostic code. However, this result has not reported this study because it came from an indirect estimation. Further studies are needed to determine the exact cancer recurrence and death in patient with cancer history.

Patients and their family will suffer from double jeopardy due to cancer and AAA and wish to understand the short- and long-term prognosis [22]. In addition to reporting the poor long-term survival of AAA repair, our study identified a history of cancer as a significant risk factor [8, 23]. Although there is no difference in perioperative mortality rate, a poor long-term survival would be disappointing news for the patients.

## Conclusion

Malignancy in AAA patients can complicate the disease course and treatment as well as outcome. History of cancer increases long-term mortality after AAA repair, but there is no difference in short-term mortality after both OSR and EVAR. Well-planned OSR and EVAR can be safely performed even in patients with intact AAA and history of intra-abdominal cancer. However, in patients with history of lung cancer, AAA repair could increase both short- and long-term mortality, and therefore, cases should be carefully selected.

## Data Availability

The datasets analyzed during the current study are available from the corresponding author on reasonable request.

## Abbreviations

AAA: Abdominal aortic aneurysm
CCI: Charlson Cormorbidity Index
CI: Confidence intervals
COPD: Chronic obstructive pulmonary disease
ESRD: End-stage renal disease
EVAR: Endovascular aortic repair
HIRA: Health Insurance Review and Assessment Service
HR: Hazard ratios
ICD-10: International Classification of Diseases, Tenth Revision
OSR: Open surgical repair
